# Long-Term Hearing, Language, and Educational Outcomes After Cochlear Implantation in Children With Waardenburg Syndrome

**DOI:** 10.7759/cureus.97615

**Published:** 2025-11-23

**Authors:** Sayaka Arai, Ryota Tomioka, Kyoko Shirai, Nobuhiro Nishiyama, Keitaro Miyake, Teruhisa Yano, Kiyoaki Tsukahara

**Affiliations:** 1 Otolaryngology - Head and Neck Surgery, Tokyo Medical University, Tokyo, JPN

**Keywords:** auditory perception, cochlear diseases, cochlear implants, hearing aids, sensorineural hearing loss, speech discrimination tests, waardenburg syndrome

## Abstract

Objective

The objective of this study was to evaluate hearing and language outcomes in children with Waardenburg syndrome (WS) who underwent cochlear implantation (CI) at a single center in Japan.

Methods

A retrospective review was performed on 12 children with WS who received CI between 1995 and 2020. Preoperative and postoperative auditory thresholds, speech recognition and language comprehension scores, and school placement were analyzed. Outcomes were compared according to the timing of intervention and the presence of cochlear malformations.

Results

The mean age at CI was 2.5 years. Early intervention was correlated with higher 67S and CI-2004 word/sentence scores. Children without cochlear malformations or delayed treatment achieved mean speech recognition scores exceeding 80%. All participants advanced to regular junior high school classes.

Conclusion

Early CI in children with WS contributes to substantial improvements in hearing, language comprehension, and educational integration.

## Introduction

Waardenburg syndrome (WS) is a genetic disorder resulting from the abnormal localization of neural crest cells, including melanocytes. Although some manifestations are cosmetic, such as a white forelock and lateral displacement of the inner canthus, congenital sensorineural hearing loss is a major clinical feature. Congenital hearing loss associated with WS accounts for approximately 1-3% of childhood hearing loss. WS is classified into four types (I-IV), with types I and II being the most common. Type III is additionally characterized by upper limb skeletal muscle abnormalities, whereas type IV is associated with Hirschsprung's disease. To date, six causative genes have been identified, and the reported penetrance of hearing loss in type I is relatively low, at 57-58%. Evaluating cochlear implant (CI) hearing performance following the diagnosis of hearing loss is essential for informed decision-making regarding CI in WS. Although the usefulness of CI for WS hearing loss has been reported in Japan and internationally, only a few Japanese reports exist, and long-term outcomes remain insufficiently documented. Therefore, this study aimed to examine the hearing outcomes after CI in patients with WS treated at our hospital. The aim of this study was to evaluate hearing, language, and educational outcomes following cochlear implantation in children with Waardenburg syndrome. We also sought to examine whether factors such as age at implantation and the presence of inner ear malformations were associated with postoperative outcomes.

## Materials and methods

Study design

This study was a retrospective observational study conducted at Tokyo Medical University Hospital. The study adhered to the STrengthening the Reporting of OBservational studies in Epidemiology (STROBE) guidelines for reporting observational studies.

Setting

Medical records of pediatric patients with Waardenburg syndrome (WS) who underwent cochlear implantation (CI) between January 2005 and December 2020 were reviewed. All audiological and language assessments were performed at the same tertiary referral center.

Participants

Children were eligible if they: 1) had a genetically or clinically confirmed diagnosis of WS; 2) presented with severe-to-profound congenital sensorineural hearing loss; 3) underwent unilateral or bilateral CI during the study period; and 4) had available preoperative or postoperative audiological records

Exclusion criteria included incomplete audiological or language assessment data and insufficient clinical records.

A total of 12 children met the inclusion criteria. Seven underwent simultaneous bilateral CI. No cases were excluded due to loss to follow-up.

Variables

The primary outcomes were CI-aided hearing thresholds and speech perception scores, including the Japanese 67S single-syllable test and CI-2004 word and sentence recognition tests.

Secondary outcomes included cognitive performance (WISC-IV) and educational placement (kindergarten through high school).

Predictor variables included age at CI, age at hearing loss diagnosis, duration of unaided hearing loss, presence of inner-ear malformations, and a family history of WS or hearing loss.

Data sources and measurement

Hearing outcomes were assessed using the Japanese 67S single-syllable speech perception test and the CD speech discrimination scoring system (CI-2004 test) for word and sentence recognition, both related to school-attending hearing ability. Speech perception was evaluated using the Japanese 67S single-syllable speech perception test [1] and the CI-2004 word and sentence recognition tests [2], both developed by the Japan Audiological Society. The verbal comprehension index and vocabulary subtest were administered by a certified clinical psychologist in a quiet environment. All speech perception tests (67S and CI-2004) were performed in a soundproof booth at 65 dB SPL using the participant's regular speech processor settings. Cognitive function was assessed using the Wechsler Intelligence Scale for Children, Fourth Edition (WISC-IV) [3]. Permission for the use of the Wechsler Intelligence Scale for Children (WISC) was obtained from Pearson Education. 

High-resolution temporal bone CT and internal auditory canal MRI were used to evaluate inner-ear malformations.

Bias

Potential biases included missing data, heterogeneity in WS subtypes, and variability in age at implantation. To minimize measurement bias, all audiological tests were performed by the same experienced audiologists using uniform procedures. Data extraction was performed independently by two investigators.

Study size

No formal sample size calculation was performed because all eligible WS patients treated at the institution during the study period were included.

Statistical analysis

Continuous variables were summarized as means and standard deviations. Group comparisons were conducted using Student's t-test or the Mann-Whitney U test, depending on data distribution. Correlation analyses between background variables and speech perception scores were performed using Pearson correlation coefficients. Statistical significance was defined as p<0.05. Analyses were conducted using SPSS version 26 (IBM Corp., Armonk, NY, USA).

Ethics

The study was approved by the Ethics Committee of Tokyo Medical University (approval no. T2020-0364). Written informed consent for data use and publication was obtained from all parents or legal guardians.

## Results

Participant flow and characteristics

A total of 12 children with genetically or clinically confirmed Waardenburg syndrome (WS) who underwent cochlear implantation (CI) were included in the analysis. No eligible patients were excluded due to missing data or loss to follow-up.

Baseline demographic and clinical characteristics are summarized in Table 1. Of the 12 participants, nine (75%) had WS type I, two (17%) had type II, and one (8%) had type IV. Inner-ear malformations were identified in three patients (25%), and one patient (8%) had intellectual disability. Six patients (50%) had a positive family history of WS or hearing loss.

**Table 1:** Audiometry, speech recognition, and language comprehension scores ※Not tested is indicated by - Bilateral test scores are indicated as right/left/both VIQ/VCI - verbal intelligence quotient/verbal comprehension index; PIQ/PRI - performance intelligence quotient/perceptual reasoning index; HA - hearing aid; CI - cochlear implantation The mean preoperative aided threshold was 71.0 ± 14.8 dB, and the mean postoperative CI-aided threshold was 32.6 ± 9.9 dB. The 67S single-syllable recognition test showed a mean score of 78.9 ± 21.6%, while the CI-2004 word and sentence recognition scores were 86.7 ± 27.6% and 72.3 ± 12.1%, respectively. Cognitive assessment revealed a mean verbal intelligence quotient/verbal comprehension index (VIQ/VCI) of 81.3 ± 16.9 and a mean performance intelligence quotient/perceptual reasoning index (PIQ/PRI) of 101.6 ± 18.6.

Case	HA threshold (dB)			CI threshold (dB)			67S (%)			CI-2004 word (%)			CI-2004 sentence (%)			VIQ/VCI	PIQ/PRI
	Right	Left	Both	R	L	B	R	L	B	R	L	B	R	L	B		
1	81	110	-	30	30	-	35	15	-	-	-	4	-	-	0	68	126
2	69	-	-	30	-	-	95	-	-	100	-	-	88	-	-	94	101
3	-	64	-	-	32	-	-	80	-	-	96	-	-	94	-	79	94
4	61	70	51	30	-	-	-	-	95	-	-	96	-	-	69	72	94
5	70	59	71	26	-	-	80	10	-	-	-	92	-	-	65	62	67
6	95	-	-	24	-	-	100	-	-	100	-	-	65	-	-	84	127
7	43	60	59	33	29	-	90	75	100	-	-	96	-	-	87	107	93
8	48	-	-	26	-	-	90	-	-	100	-	-	100	-	-	107	111
9	61	68	65	20	22	20	85	80	95	100	92	-	94	75	-	-	-
10	86	-	-	42.5	-	-	-	-	-	-	-	-	-	-	-	-	-
11	-	-	50	40	37.5	40	40	30	35	-	-	52	-	-	-	-	-
12	88	41	-	56	25	20	-	-	-	-	-	-	-	-	-	-	-

The mean age at hearing-loss diagnosis was 3.0 ± 2.0 months, and the mean age at hearing-aid fitting was 6.9 ± 5.3 months. The mean age at implantation was 30.4 ± 13.8 months. Seven children (58%) underwent simultaneous bilateral CI.

Hearing outcomes

**Figure 1:**
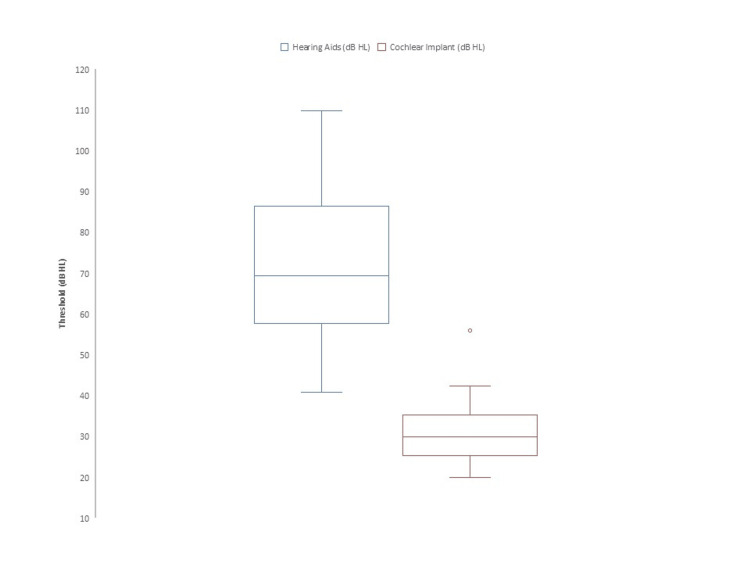
Aided hearing thresholds (dB) Postoperative hearing thresholds decreased in all cases compared with preoperative values. Values are presented as mean ± SD (preoperative: 71.0 ± 14.8 dB; postoperative: 32.6 ± 9.9 dB).

Audiometric outcomes are summarized in Table 2. The mean preoperative aided pure-tone threshold was 71.0 ± 14.8 dB. After implantation, the mean CI-aided threshold improved to 32.6 ± 9.9 dB. Hearing thresholds improved in all patients (Figure 1).

**Table 2:** Patient background The study included 12 children (nine with type I (75%), two with type II (17%), and one with type IV (8%) Waardenburg syndrome). A positive family history of hearing loss or WS was found in six patients (50%), and inner-ear malformations were identified in three patients (25%). One patient (8%) had intellectual disability. Values are presented as N (%) for categorical variables and as mean ± standard deviation (SD) for continuous variables. Mean age at cochlear implantation: 30.4 ± 13.8 months (2.5 ± 1.2 years); mean age at hearing aid use: 6.9 ± 5.3 months (0.6 ± 0.4 years). WS - Waardenburg syndrome; HA - hearing aid; CI - cochlear implantation

Case	WS type	Family history	Hearing loss diagnosis age	HA use age	CI side	Complications	CI age
1	I	None	1y 9m	1y 9m	Bilateral		5y 3m
2	I	Maternal second cousin: hearing loss	3m	6m	Right		2y 2m
3	II	Father and sister: WS, hearing loss, heterochromia iridum	1m	3m	Left		3y 10m
		Uncle: hearing loss					
4	I	Paternal grandfather: hearing loss	3m	5m	Bilateral		3y 1m
5	I	None	0m	5m	Bilateral	Bilateral enlarged vestibular aqueduct	1y 7m
6	I	None	3m	4m	Right		2y 2m
7	I	None	0m	6m	Bilateral		1y 5m
8	I	Parents: deaf	0m	8m	Right		2y 9m
		Sister: muscular dystrophy					
9	I	Mother: heterochromia iridum, hearing loss	0m	8m	Bilateral		1y 10m
10	II	Mother: WS	0m	3m	Right	Mental retardation	2y 2m
		Father: hearing loss					
11	IV	None	4m	9m	Bilateral	Left cochlear hypoplasia (1.5 turns)	2y 11m
12	I	None	1m	4m	Bilateral	Right internal auditory canal stenosis	1y 2m

Speech perception outcomes

**Figure 2:**
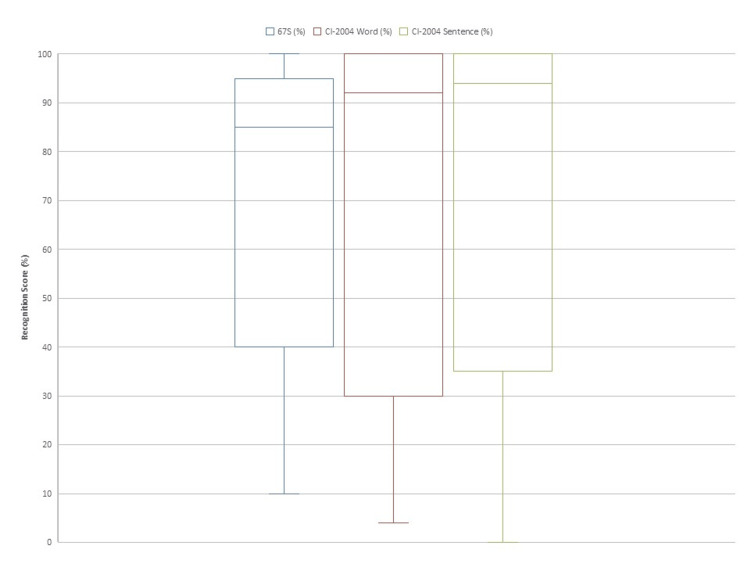
Speech recognition scores (%) Boxplots for 67S single-syllable, CI-2004 word, and CI-2004 sentence recognition scores. Mean scores were 78.9 ± 21.6%, 86.7 ± 27.6%, and 72.3 ± 12.1%, respectively.

The mean 67S single-syllable recognition score was 78.9 ± 21.6%. The mean CI-2004 word and sentence recognition scores were 86.7 ± 27.6% and 72.3 ± 12.1%, respectively (Figure 2).

Two cases (case 1 and case 11) demonstrated lower speech perception scores compared with the cohort. Case 10 and case 12 were evaluated only using CI-aided thresholds because standardized speech perception tests could not be completed due to developmental or anatomical factors.

Cognitive outcomes

WISC-IV scores were available for eight children (66%). The mean verbal comprehension index (VCI) was 81.3 ± 16.9, and the mean perceptual reasoning index (PRI) was 101.6 ± 18.6. Two children had not yet reached school age and did not undergo cognitive testing.

Educational outcomes

School placement data were available for 10 children. Eight children (80%) were enrolled in regular elementary and junior high school classes, while two attended schools for the deaf. All children with available data advanced to regular junior high school classes. Table 3 details educational progression from kindergarten to higher education.

**Table 3:** School type Eight of ten patients (80%) were enrolled in regular elementary and junior high school classes, while two (20%) attended schools for the deaf.

Case	Kindergarten	Elementary school	Junior high school	High school	University
1	Deaf and regular	Regular	Regular	Regular	Regular
2	Deaf	Regular	Regular	Regular	Regular
3	Deaf	Regular	Regular	Deaf	
4	Regular	Regular	Regular	Regular	Regular
5	regular	Regular	Regular	Regular	
6	Deaf	Regular	Regular		
7	Deaf	Regular	Regular	Regular	
8	Regular	Regular			
9	Deaf	Unidentified			
10	Deaf				
11	Deaf	Deaf	Regular		
12	Unidentified				

Correlation analyses

Table 4 presents correlations between background factors and auditory outcomes. Key findings include: age at CI was significantly correlated with 67S scores (p=0.002), CI-2004 word scores (p=0.004), and sentence scores (p=0.018); duration of hearing loss (diagnosis to CI) significantly correlated with 67S (p<0.001) and CI-2004 word scores (p=0.001); aided thresholds before CI were correlated with postoperative CI-aided thresholds (p<0.001); and age at diagnosis showed significant correlations with all speech recognition outcomes (p<0.001).

**Table 4:** Correlation between background factors and speech recognition scores Statistical analysis was performed using the Student's t-test. A p-value of <0.05 was considered statistically significant. Values indicate p-values; t-values are shown in parentheses. * p<0.05 Hearing loss period: the period from hearing loss diagnosis until CI HA - hearing aid; CI - cochlear implantation

	CI aided threshold (dB)	67S (%)	CI-2004 W (%)	CI-2004 S (%)
Aage at CI (month)	0.606 (-0.531)	0.002* (-4.263)	0.004* (-3.766)	0.018* (-2.984)
Hearing loss period (months)	0.246 (-1.227)	< 0.001* (-5.318)	0.001* (-4.658)	0.005 (-3.822)
HA threshold (dB)	< 0.001* (7.860)	0.14 (-1.619)	0.153 (-1.562)	0.639 (-0.487)
Age at diagnosis (months)	< 0.001* (-7.542)	< 0.001* (-8.270)	< 0.001* (-6.768)	< 0.001* (-5.697)

Comparison with other etiologies

Table 5 compares WS outcomes with those of non-syndromic congenital hearing loss, including enlarged vestibular aqueduct (EVA). Among WS patients without inner-ear malformations or delayed intervention, mean auditory performance (67S: 81.5 ± 17.4%; CI-2004 word: 92.4 ± 15.7%; sentence: 84.0 ± 11.6%) closely approximated the outcomes observed in non-syndromic patients (67S: 83.5 ± 12.2%). WS cases with cochlear hypoplasia or cochlear canal stenosis demonstrated poorer performance.

**Table 5:** Comparison of speech recognition score means in uncomplicated WS, enlarged vestibular aqueduct WS, overall WS cases, and WS cases excluding complications and delayed therapeutic intervention at our hospital Congenital hearing loss CI surgery example at our hospital. No complications: surgery under four years of age, excluding non-syndromic hearing loss, undiagnosed cases of hearing loss, inner ear malformations, and developmental delays WS - Waardenburg syndrome; VIQ/VCI - verbal intelligence quotient/verbal comprehension index; PIQ/PRI - performance intelligence quotient/perceptual reasoning index; CI - cochlear implantation

Group	n	CI threshold (dB)	67S (%)	CI-2004 word (%)	CI-2004 sentence (%)	VIQ/VCI	PIQ/PRI
No complications	257	29.4 ± 8.7	83.5 ± 12.2	90.5 ± 10.8	83.3 ± 11.3	88.2 ± 14.5	103.7 ± 13.4
WS all	19	30.8 ± 9.9	68.3 ± 21.6	84.4 ± 27.6	73.7 ± 12.1	84.1 ± 16.9	101.6 ± 18.6
WS without complications/delayed intervention	15	27.8 ± 8.3	81.5 ± 17.4	92.4 ± 15.7	84.0 ± 11.6	87.3 ± 13.5	106.7 ± 14.9
Enlarged vestibular aqueduct	22	26.8 ± 9.2	85.8 ± 13.1	89.3 ± 14.5	86.3 ± 11.9	100.0 ± 12.3	111.3 ± 10.8

## Discussion

Histologically, hearing loss associated with WS is primarily attributed to inner-ear damage, including the loss of the organ of Corti and atrophy of the stria vascularis. Consequently, CI often yields favorable outcomes. However, nerve fiber atrophy and loss of spiral ganglion cells have also been reported, which may contribute to poor hearing. In all CI cases treated at our hospital, auditory thresholds improved from the preoperative hearing aid thresholds to the CI-aided thresholds. The 67S and CI-2004 word and sentence recognition scores were also higher in patients without cochlear malformations or delayed intervention. Overall, language comprehension scores were within the average range for cognitive development.

Several studies have demonstrated the efficacy of CI in WS [4,5]. Significant improvements in perceptual ability and speech intelligibility have been reported in all patients without malformations [6] and even in those with cochlear incomplete partition type II deformities [7]. Our findings similarly showed that the CI-aided threshold was lower than the preoperative aided threshold in all cases, suggesting that CI may be effective for children with WS-associated hearing loss. Previous reports have also indicated that postoperative auditory performance in CI recipients with WS is comparable to that of CI recipients without syndromic hearing loss [8,9]. In this study, patients’ speech recognition scores were particularly high in patients without cochlear deformities, consistent with the findings of previous reports.

With CI, children with severe hearing loss can acquire spoken language through auditory input and often progress to regular schools [10]. Globally, children with hearing impairment are frequently enrolled in regular schools [11], and one study reported that 95% of 42 children with CIs attended regular schools [12]. Our results likewise showed that most elementary and junior high school-aged children attended regular schools. Although treatment protocols and surgical indications vary across countries, our findings are comparable to those reported in patients without WS, suggesting that CI may positively influence quality of life and school placement in WS.

Nonetheless, some patients demonstrated poor outcomes in speech recognition and language comprehension tests. Contributing background factors included delayed intervention, intellectual disability, incomplete electrode insertion due to cochlear hypoplasia of the cochlea, and cochlear canal stenosis. These conditions may account for suboptimal postoperative auditory performance. Among pediatric CI recipients with cochlear malformations according to Jackler's system, CI performance is generally maintained across malformation types. However, auditory perception and language development outcomes are more favorable in cases involving incomplete partitions than in those involving common cavity deformities or cochlear hypoplasia, and extremely poor speech and language performance have been reported in cases with cochlear nerve deficiency [13,14]. Furthermore, CI has been shown to restore functional hearing in cases with enlarged vestibular aqueducts [15].

These considerations generally suggest that patients with WS who may have cochlear malformations also require careful preoperative imaging studies. When such malformations are identified, the potential for poor postoperative outcomes should be anticipated and clearly explained to the patient. Although some patients with WS type I lack malformations [16], caution is warranted, as semicircular canal abnormalities, enlarged vestibules, reduced cochlear size, abnormal morphology, and cochlear nerve defects have been reported in patients with SOX10 mutations [17]. Therefore, early confirmation of the presence or absence of inner ear malformations through imaging, along with genetic testing, is essential. In this study, audiometric results were relatively favorable in case five, which presented with a dilated vestibular aqueduct, but poorer in case 11, involving cochlear hypoplasia, and in case 12, with a narrowed cochlear canal. These findings are consistent with those of the aforementioned studies and suggest that hearing deterioration due to malformations may occur in WS, similar to that seen in general forms of sensorineural hearing loss. Case 10 demonstrated a low CI-aided threshold relative to age and was assessed solely on this basis. CI intervention may be delayed in individuals with intellectual disability, cognitive impairment, or learning difficulties compared with CI for general hearing loss [18]. Early preoperative identification of such comorbidities may therefore help predict CI effectiveness. In case one, the intervention for hearing loss was delayed, and the CI intervention was performed too late. The critical period for auditory acquisition of spoken language occurs around 2.5 years of age [19]. In children with congenital hearing loss, earlier hearing aid use is associated with improved subsequent language development [20-22], and initiation within three months of age correlates with improved language expression and comprehension [20]. Earlier CI has also been linked to superior speech and language performance [23,24], with increasing evidence supporting CI before one year of age [25,26]. Thus, delays in intervention for hearing loss, both in children with WS and those with non-syndromic hearing loss, may negatively affect postoperative auditory outcomes. Early initiation of hearing aid use and CI is therefore desirable in WS cases, as well as in general severe hearing loss cases. Earlier diagnosis was also correlated with all speech recognition scores in relation to background factors. In addition, earlier age at surgery and duration of hearing loss were correlated in some cases. In our previous study [27] of adults without cochlear malformations, a strong correlation was observed between the hearing aid use threshold and the duration of hearing aid use. Therefore, examining a larger population of WS cases would be valuable for further comparison.

The penetrance of WS features varies widely [28,29], and many affected children may remain undiagnosed. Because the prevalence of sensorineural hearing loss in WS type I is relatively low [30], genetic testing should be considered when a child is diagnosed with congenital hearing loss. Early identification of WS enables timely intervention with hearing aids or CI, maximizing developmental and communicative outcomes.

Our findings are consistent with previous studies showing that early implantation and normal inner ear anatomy are associated with better speech and educational outcomes in children with Waardenburg syndrome [7,10,12]. Conversely, delayed implantation and additional disabilities have been reported to result in poorer performance [13,15], which aligns with our cases showing limited improvement (cases 1, 11, and 12). This study has several limitations. The small sample size and heterogeneous characteristics of the cohort, including different WS subtypes and cognitive profiles, may limit the generalizability of our findings. Therefore, these results should be interpreted with caution. In addition, as this was a single-center retrospective study, multicenter prospective studies with more homogeneous populations and standardized language assessments are needed to confirm our results.

## Conclusions

We retrospectively reviewed 12 cases of clinically diagnosed WS with hearing loss treated with CI. Postoperatively, most patients achieved relatively high hearing and language performance by school entry and were enrolled in regular classes.

Patients with WS exhibit few serious complications other than hearing loss, suggesting that early recognition of characteristic clinical features, timely diagnosis, and prompt auditory intervention can markedly improve quality of life. Further long-term follow-up studies with larger cohorts are warranted.
